# Refining MMA Screening in the Dutch Newborn Screening Program: Lessons from Vitamin B12 Deficiency and Genetic Cases

**DOI:** 10.3390/ijns12030069

**Published:** 2026-08-21

**Authors:** Nils W. F. Meijer, Klaas Koop, Rose E. Maase, Patricia L. Hall, Wouter F. Visser, Esmeralda Oussoren, Annet M. Bosch, M. Rebecca Heiner-Fokkema, Monique G. M. de Sain-van der Velden

**Affiliations:** 1 Department of Genetics, Section Metabolic Diagnostics, University Medical Center Utrecht, Lundlaan 6, 3584 EA Utrecht, The Netherlands; 2 Reference Laboratory for Neonatal Screening, Center for Health Protection, National Institute for Public Health and the Environment (RIVM), 3721 MA Bilthoven, The Netherlands; 3 Department of Laboratory Medicine and Pathology, Mayo Clinic, Rochester, MN 55905, USA; 4 Department of Pediatrics, Center for Lysosomal and Metabolic Diseases, Erasmus MC University Medical Center, 3015 GD Rotterdam, The Netherlands; 5 Division of Metabolic Diseases, Department of Pediatrics, Emma Children’s Hospital, Amsterdam Gastroenterology Endocrinology and Metabolism, University of Amsterdam, Amsterdam UMC, Meibergdreef 9, 1105 AZ Amsterdam, The Netherlands; 6 Laboratory of Metabolic Diseases, Department of Laboratory Medicine, University of Groningen, University Medical Center Groningen, 9700 RB Groningen, The Netherlands

**Keywords:** methylmalonic acidemia, methylmalonic acid, CLIR, vitamin B12 deficiency, newborn screening

## Abstract

Since October 2019, screening for methylmalonic acidemia (MMA) has been implemented in the Dutch Newborn Screening (NBS) program. Since implementation, most referrals for MMA have been the result of (maternal) vitamin B12 deficiency. Although vitamin B12 deficiency is an important condition and early detection can confer health benefits, its identification was not the original objective of the screening. We therefore examined whether genetic forms of MMA can be distinguished from acquired forms. Early distinction between these forms is essential for guiding clinical management, informing prognosis, and enabling appropriate genetic counseling. Specifically, we tested whether the use of Collaborative Laboratory Integrated Reports (CLIR) to complement NBS results improved specificity of the test for genetic MMAs. We found that with the use of CLIR, genetic conditions including methylmalonyl-CoA mutase (mut) deficiency and cobalamin (cbl) A, B, C and D deficiency can be partially differentiated from acquired causes. This results in a significant reduction in the number of second-tier tests required. However, this approach may also exclude certain other genetic causes. Based on all findings, we provide considerations and implications for MMA screening that may inform decision-makers in (other) NBS programs.

## 1. Introduction

Methylmalonic acidemia (MMA) can originate from inherited or acquired causes. Genetic forms result from defects in enzymes involved in methylmalonyl-CoA metabolism or in its cofactor, adenosylcobalamin (for example, variants in *MUT* [ENSG00000146085], *MMAB* [ENSG00000139428], or *MMACHC* [ENSG00000132763]). Acquired forms are typically associated with vitamin B12 deficiency, renal dysfunction, or prematurity [1,2,3,4,5,6,7]. In most cases, early detection of genetic causes of MMA enables intervention to mitigate harm, and it is therefore included in newborn screening (NBS) programs. Referrals from NBS programs are generally based on elevated concentrations of methylmalonic acid (MMA)^MB^, which serves as a second-tier NBS marker [8]. Throughout the manuscript, “MMA” refers to methylmalonic aciduria (the disease), whereas MMA^MB^ denotes methylmalonic acid, the metabolite.

The clinical presentation of “genetic MMAs” can vary widely depending on the subtype and specific variant [9,10]. In contrast, acquired MMA typically reflects transient metabolic disturbances rather than permanent enzymatic defects. Unlike genetic forms, acquired MMA is usually reversible once the underlying cause is identified and addressed. Failure to distinguish between these etiologies during NBS may result in unnecessary diagnostic procedures, inappropriate treatment strategies, and avoidable anxiety for patients and their families. Therefore, early and accurate differentiation supports targeted intervention, informed decision-making, and optimal patient care. Leveraging NBS data to distinguish between genetic and acquired causes may support this differentiation.

MMA was added to the Dutch NBS program in 2019, and the screening process is based on a two-tiered approach. In the first tier, C3 carnitine and the ratios C3/C2 and C3/C16 are determined using the Revvity Neobase^TM^ 2 Non-derivatized kit and the Waters Xevo TQD analysis platform. If first-tier marker and marker ratios are elevated, a second-tier analysis is conducted to measure MMA^MB^ (multiplexed with methylcitric acid, MCA). The second-tier method is an in-house LC-MS/MS method, as no commercial test kits are currently available for the determination of MMA^MB^ in dried blood spots (DBS) [11]. The first-tier marker and marker ratios can be elevated due to a variety of causes other than MMA, including kidney dysfunction, vitamin B12 deficiency or prematurity [2,3,4,12], and the second tier is essential to improve the specificity of the NBS and reduce unnecessary referrals. Hence, MMA^MB^ provides greater specificity for detecting MMA. When the diagnosis of (maternal) vitamin B12 deficiency cannot be established with certainty, additional diagnostic investigations, including genetic testing, are performed to establish the final diagnosis.

Since the implementation of NBS for MMA in the Netherlands, the number of referrals for MMA has been higher than expected. Evaluations indicate that a large proportion (approximately 80%) of these referrals have been related to (maternal) vitamin B12 deficiency. Nonetheless, from a qualitative perspective, MMA screening performs well: the elevated MMA^MB^ measured within the NBS program has, for each referred neonate, been confirmed by diagnostic follow-up, and clinical, treatable causes for these elevations have been identified, with no evidence of missed true positive cases. It is for these reasons that MMA screening has remained unchanged since 2019 despite the higher-than-expected number of referrals [13].

The aim of this study is to retrospectively analyze NBS data to determine whether optimization of MMA NBS can be achieved by (i) evaluating whether the use of Collaborative Laboratory Integrated Reports (CLIR) enables the segregation of the different causes of elevated MMA^MB^, to provide clinicians with information on the most likely underlying form of MMA at the time of referral or (ii) modifying the first-tier algorithm to decrease the number of samples for which second-tier testing is required.

## 2. Materials and Methods

### 2.1. The Study Population

The study population consisted of referrals for MMA (reference MMA^MB^ ≥ 5 µmol/L) for which the underlying cause of elevated MMA^MB^ was determined during the period 10 January 2019 to 31 December 2024. Data were obtained from the Dutch National Institute for Public Health and the Environment, Department for Vaccine Supply and Prevention Programs (RIVM-DVP) registry. This study was approved by the Data Applications Committee of Praeventis at RIVM-DVP. Each dataset comprised first- and second-tier marker concentrations as well as biological sex, gestational age, birthweight and age at heel prick. Data were included only from neonates whose parents did not object (before 2023) or consented (since 2023) to the use of heel-prick data for research purposes. The dataset was pseudonymized before data analysis. Information on the diagnosis for each case was obtained from the Dutch Diagnosis Registration Metabolic Diseases (DDRMD) and cases included: transcobalamin I (TC1) carrier (*n* = 1), B12 deficiency (*n* = 34), CD320 deficiency (*n* = 3), cblC carrier (*n* = 2), cblC (*n* = 1) and mut- (*n* = 1). Prior to publication, written informed consent was obtained from the parents/legal guardians of all patients with a genetic form of MMA.

### 2.2. Data Selection and Inclusion

During the study period, a small subset of neonates were screened before the target window (<3 days of age). In this group, the heel prick was obtained early for several reasons, such as the need for blood transfusion or administration of medication. Consequently, it is reasonable to assume that this subset may include severely ill newborns, which could impact the generalizability of the study results. For this reason, the study population was restricted to samples from neonates between 3 and 183 days of age. This extended period reflects the policy in which infants are offered NBS up to the age of 6 months. Additional inclusion criteria included a birthweight between 500 and 6000 g and a gestational age in the range of 161–308 days (23–44 weeks).

### 2.3. Collaborative Laboratory Integrated Reports

Collaborative Laboratory Integrated Reports (CLIR) is a multivariate pattern recognition software coupled to an interactive database comprising international screening data: reference (normal, “healthy” population) data as well as false- and true-positive cases for specific conditions [14]. Datasets in the database contain analytical data (screening marker values) and the covariates biological sex, gestational age, birthweight, age at heel prick and Julian date. The CLIR post-analytical tools can be utilized to examine the data by application of different functionalities. The productivity tools contain various means to visualize patterns within the data such as the effects of covariates or the relationship between different markers. Alternative tools specific to conditions of interest can be generated to define scores that represent the likelihood of a datapoint belonging to a specific condition category.

For this study the “Condition versus Condition” tool functionality was utilized to define and represent markers that best distinguish genetic and acquired forms of MMA in CLIR by leveraging, for each case, all markers included in the NeoBase^TM^ 2 Kit. Currently, CLIR includes the following MMA condition categories: methylmalonic acidemia (abbreviation: MUT/Cbl AB; containing mutase, cblA and cblB cases; *n* = 228), methylmalonic acidemia Cbl F (abbreviation: MMA Cbl F; containing: cblF cases; *n* = 7), methylmalonic aciduria and homocystinuria (abbreviation: Cbl CD; containing: cblC and cblD cases; *n* = 177), transcobalamin receptor defect (abbreviation: TCblR; containing TCblR cases, also referred to as CD320 deficiency; *n* = 14) and maternal vitamin B12 deficiency (abbreviation: B12 Def (mat); containing vitamin B12 deficiency (as a consequence of maternal B12 deficiency) cases; *n* = 204). Hence, either the MUT/Cbl AB category or Cbl CD category was compared against the B12 def (mat) category as the basis for our analysis (settings displayed in Table 1), resulting in the markers displayed in Table 1. The MMA Cbl F category (*n* = 7) and TCblR category (*n* = 14) were excluded due to the limited number of cases available in CLIR, which does not adequately capture the variability within these condition-specific populations.

Next, the “Plot by Condition” functionality (settings included: preferred adjustments: Age, BW, and location; values in the plot are visualized as Z-scores) was used to visualize the covariate corrected intervals for each of the obtained discriminative markers in each condition (Figure 2B).

Discriminative markers for each condition category (MUT/CblAB and Cbl CD) were then utilized to develop “Single Condition Tools”(SCT) to calculate scores for each positive MMA screening result with a clear genetic diagnosis. Details of the SCT configuration for MUT/Cbl AB and Cbl CD are provided in Table 2.

## 3. Results

### 3.1. Evaluation of C3, MMA^MB^ and the C3/C2 Ratio in DBS for Differentiating Acquired and Genetic Causes

**Figure 1:**
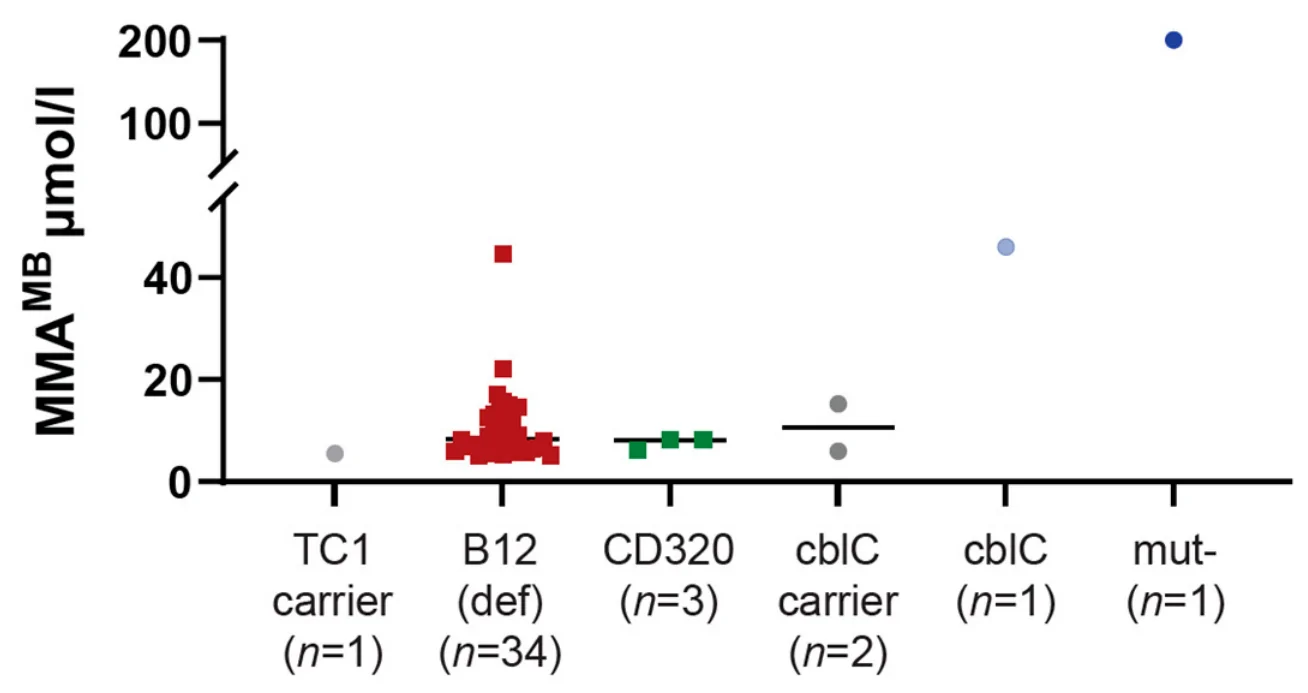
Scatterplot depicting MMA^MB^ concentration in genetic and acquired forms of MMA detected via the Dutch MMA NBS (2019–2024).

To determine if C3, MMA^MB^ concentration or the C3/2 ratio in DBS can serve as a reliable differential predictor of disease and conditions associated with high MMA^MB^, Dutch referrals for MMA for which the underlying cause was determined through clinical follow-up were examined. This included patients with transcobalamin I (TC1) carrier (*n* = 1), B12 deficiency (*n* = 34), CD320 deficiency (*n* = 3), cblC carrier (*n* = 2), cblC (*n* = 1) and mut- (*n* = 1). Within this subset, C3 and MMA^MB^ concentrations ranged from 1.87 to 9.88 µmol/L and from 5.06 to 200 µmol/L. For both markers, values overlapped across all categories (Figure 1 and Supplementary Figure S1A). With regard to the C3/C2 ratio, higher concentrations were found for cblC and mut- as compared to cases with vitamin B12 deficiency. While this might suggest that the C3/C2 ratio differentiates between these conditions, it is primarily due to the limited amount of case data available. When considering the full range of each condition-specific interval, there is a clear overlap between the three conditions (Supplementary Figure S1B). These results therefore show that neither the C3 or the MMA^MB^ concentration nor the C3/C2 ratio enable differentiation between acquired and genetic forms of MMA.

### 3.2. Computational Differentiation Between Acquired and Genetic Forms of MMA Using CLIR

**Figure 2:**
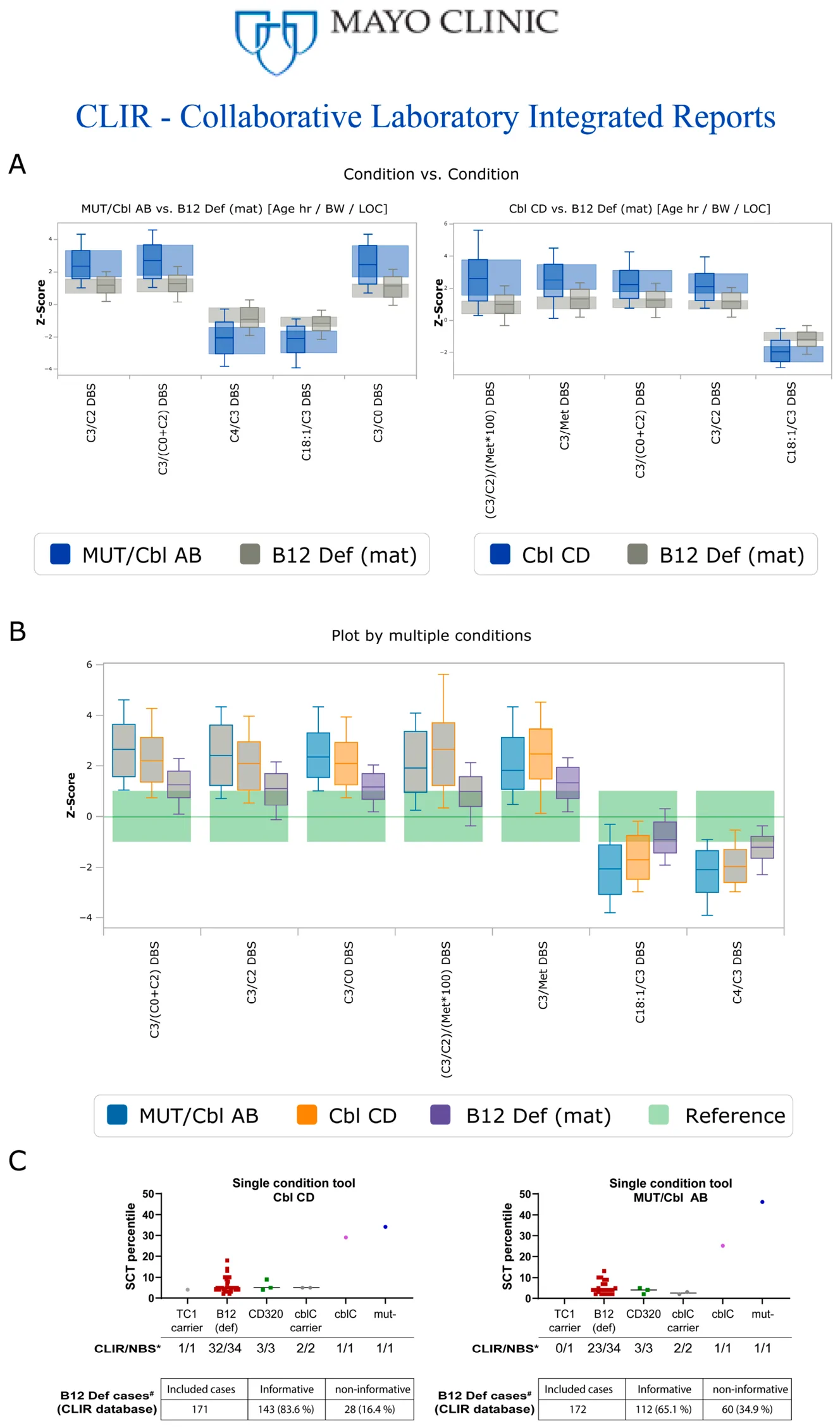
(**A**) Markers that differentiate between either the mut/cbl AB or the cbl CD and the maternal vitamin B12 deficiency category based on the “Condition vs. Condition” tool in CLIR (settings included: percentage of cases: 80 and a maximum overlap range of 22% for the mut/cbl AB comparison and 32% for the cbl CD comparison). (**B**) Plot by Multiple Conditions depicting the C3/(C0+C2) ratio, C3/C2 ratio, C3/C0 ratio, (C3/C2)/(Met*100) ratio, C3/Met ratio, C18:1/C3 ratio and the C4/C3 ratio in methylmalonic acidemia (mut/cbl AB), methylmalonic aciduria and homocystinuria (cbl CD) and maternal vitamin B12 deficiency (B12 Def (mat). (**C**) Scatterplot indicating the informative percentiles attributed by the cbl CD and mut/cbl AB SCT to each referral for MMA. “Single Condition Tools” (SCTs) were based on the markers displayed in Figure 2B. An overview of the full list of settings can be found in Table 1 and Table 2. * Number of referrals from the Dutch NBS program versus those that would receive informative scores based on the respective SCT in CLIR. ^#^ Cases derived from the B12 Def (mat) population in CLIR. Included cases represent those containing values for all of the markers and covariates featured in the tool. Cases missing any of this data cannot be ran in the tool and were therefore excluded from the analysis (https://clir.mayo.edu/, accessed on 22 May 2026).

As an alternative approach to using only MMA^MB^ levels, we explored whether first-tier NBS data used in conjunction with CLIR could be used to separate genetic from acquired forms of MMA. Utilizing the “Plot by Condition” functionality in CLIR, we identified C3/C2, C3(C0+C2), C3/C0, (high markers), and C4/C3 and C18:1/C3 (low markers) as the most discriminative markers for the MUT/Cbl AB category (Table 1). In contrast, C3/C2, C3(C0+C2), (C3/C2)/Met*100), C3/Met (high markers), and C18:1/C3 (low marker) were most effective in distinguishing the Cbl CD category from B12 Def (mat) category (Table 1). Comparisons were limited to these three groups as they were the only categories in CLIR with sufficient cases (Figure 2A and Table 1). Condition-specific, covariate-adjusted intervals for these markers across all included conditions in CLIR were visualized using the “Plot by Multiple Conditions” functionality (Figure 2B). Across all evaluated markers, a general trend emerged: the lower echelon of the ratios in the Cbl CD category, and to a lesser extent the MUT/Cbl AB category overlaps with the upper range of values observed in the B12 Def (mat) category. Collectively, these intervals indicate that at least partial separation between groups may be possible.

We therefore created “Single Condition Tools” for the MUT/Cbl AB and Cbl CD categories based on the marker/marker ratios depicted in Figure 2B and assigned scores to each of the 42 MMA referrals. These results, presented in Figure 2C, align with the condition-specific intervals observed in CLIR, indicating that a targeted selection aimed at identifying mut- and cbl A/B, those with the most extreme biochemical phenotype, can reduce the number of MMA referrals. Additionally, based on cumulative population data in CLIR, approximately 34.9% of B12 Def (mat) may be distinguishable from the more severe MMA phenotypes using this approach. Referrals with milder metabolic phenotypes are often assumed to represent acquired conditions, such as vitamin B12 deficiency. However, our data indicate that this group may also include variants in CD320. If a more lenient selection based on the cbl CD population is applied, only very mild forms of vitamin B12 deficiency can be distinguished. This group corresponds to the lowest part of the B12 Def (mat) interval and represents approximately 16.4% of the cumulative B12 Def (mat) population in CLIR. We therefore conclude that computational differentiation using additional markers in NeoBase^TM^ 2 kit allows exclusion of a small number of the vitamin B12 deficiencies, but not exclusively since this approach may still include cases with variants in CD320.

The current first-tier screening protocol for MMA generates approximately 1200 confirmatory second-tier tests per year, most of which return negative results. We therefore retrospectively assessed whether applying the SCT established for the cbl CD and mut/cbl AB population could reduce the number of confirmatory tests. We found that this approach resulted in a reduction of 23.8% and 54.9% using the tools for cbl CD and mut/cbl AB respectively (Table 3).

## 4. Discussion

In population-based NBS, a well-defined target disease is essential, as it provides the ethical and scientific rationale for screening and enables effective monitoring and evaluation of the NBS program. Since the implementation of NBS for MMA in the Netherlands, the majority of referrals have been for acquired forms of MMA, primarily vitamin B12 deficiency. Following an MMA referral, the underlying etiology, genetic or acquired, directly shapes parental counseling. In acquired vitamin B12 deficiency, most often due to maternal deficiency during pregnancy or breastfeeding, discussions focus on the potential for full recovery with timely supplementation of vitamin B12 [15]. Parents are reassured that, with appropriate supplementation for both the infant and the mother, neurological and hematological outcomes are typically favorable. The conversation emphasizes practical steps, such as supplementation schedules, dietary adjustments, and follow-up monitoring of vitamin B12 and MMA levels. By contrast, counseling for MMA with genetic cause emphasizes the possibility of persistent metabolic abnormalities, long-term management including rigorous treatment needs which may include dietary adjustments, injections of cobalamin and potential transplants, variable but frequently poor clinical outcomes, and implications for siblings and family planning. If inclusion in the NBS of the acquired forms is not desired, they should be (clearly) separated from the genetic group to enable appropriate referral. If separation is not possible, they should be accepted as incidental findings, or one could consider changing the target disease definition. Therefore, our aim was to evaluate to what extent acquired and genetic forms of MMA can be differentiated and whether this information could be used to optimize the screening for MMA.

MMA^MB^ and C3-carnitine concentrations proved insufficient for differentiation, as did the C3/C2 ratio. As an alternative approach, we developed SCT in CLIR to assess the discriminatory power. The SCT may be valuable when limiting screening to cbl CD, and mut/cbl AB as its use can reduce second-tier testing and associated delay in NBS result release and costs significantly. However, screening captures the infant’s metabolic status at a single point in time; deficiencies that appear mild initially may progress if untreated. Decision-making therefore requires balancing the potential loss of health benefits against cost savings from reduced testing. Here, it should be noted that vitamin B12 supplementation improves maternal and infant vitamin B12 status, yet evidence for clinically relevant maternal and child health benefits is still very uncertain [16,17]. At the same time, we recognize emerging observational evidence suggesting that NBS for vitamin B12 deficiency may reduce symptomatic infantile disease, although further studies are needed to establish its clinical benefit [18,19]. Furthermore, it is unknown to what extent other forms of genetic MMA, that should be reported as incidental findings, may be detected using this method. For example, we did not anticipate detecting CD320, which encodes the transcobalamin receptor, when starting screening for MMA. Infants with a genetic defect in CD320 typically do not develop serious medical problems or metabolic decompensation during illness, at least during early childhood [20]. Counseling for this disorder is therefore challenging. Our case data, as well as that from others [20], show that biochemical abnormalities associated with this condition rapidly normalize after enteral hydroxocobalamin administration and remain normal for at least several years after discontinuation of cobalamin therapy, raising questions about the justification for “labeling” these newborns.

Although our study is limited by the relatively small number of samples, overlap in the metabolic profiles is evident between acquired and genetic forms, and the current MMA NBS algorithm and CLIR do not reliably differentiate them. Consequently, it is necessary to decide whether the detection of B12 deficiency in the screening for MMA constitutes a problem that needs to be resolved. Vitamin B12 deficiency is a well-established cause of neurological and hematological abnormalities that can be managed effectively through supplementation [21,22]. Untreated infants may be at risk for neurological complications, developmental delay, and failure to thrive [21,22]. This could provide a rationale for the inclusion of B12 deficiency in NBS programs, either as target conditions or as (acceptable) incidental findings. However, NBS target diseases need to be established by the Dutch Health Council, which is not the case for B12 deficiency at this time. Therefore, when the Dutch MMA NBS algorithm was established, vitamin B12 deficiency was not considered as a target condition and, consequently, the current screening protocol is also not designed to capture the full spectrum of vitamin B12 deficiency.

If detection of B12 deficiency is indeed deemed problematic, prevention of vitamin B12 deficiency in pregnancy may be considered. For this we propose two alternative strategies. Firstly, supplementation of vitamin B12 in national health guidelines may be considered, analogous to the recommendation for folic acid supplementation, which is already implemented in the Netherlands. Secondly, consideration could be given to targeted screening of pregnant women, particularly those adhering to vegan or vegetarian diets, for vitamin B12 deficiency during the third trimester, comparable to existing practices aimed at detecting iron deficiency. Conversely, if detection of B12 deficiency in the screening for MMA is not considered problematic, the definition of vitamin B12 deficiency may be optimized where cases detected through MMA NBS are included, either as an “acceptable incidental finding” or as a “target condition”. As the current screening algorithm is not optimized to detect all forms (mild to severe) of vitamin B12 deficiency, the former definition (acceptable incidental finding) may be more applicable.

## Figures and Tables

**Table 1:** Discriminative markers obtained by the “Condition versus Condition plot”. Case counts per condition retrieved from https://clir.mayo.edu/ (accessed on 12 February 2026).

Condition versus Condition Plot (settings: minimum of cases = ≥ 80%; maximum overlap range = ≤ 22%)		
Condition category A	Condition category B	Discriminative markers
MUT/Cbl AB (*n* = 228)	B12 Def (mat) (*n* = 204)	High Markers C3/(C0+C2) C3/C2 C3/C0
		Low Markers C18:1/C3 C4/C3
Condition versus Condition Plot (settings: minimum of cases = ≥ 80%; maximum overlap range = ≤ 32%)		
Condition category A	Condition category B	Discriminative markers
Cbl CD (*n* = 177)	B12 Def (mat) (*n* = 204)	High Markers C3/(C0+C2) C3/C2 C3/Met (C3/C2)/(Met*100)
		Low Markers C18:1/C3

**Table 2:** Configuration of the “Single Condition Tools”for mut/cbl A/B and cbl C/D. * Marker exceptions are predefined upper- or lower-marker thresholds, above or below which no disease cases, but only false-positive cases, were observed, leading to automatic non-informative classification. ^#^ Filters are predefined upper- or lower-marker thresholds beyond which values are excluded from interval calculation. Filters can be based on percentiles, interquartile range or values.

Condition Category	Configuration	Included Markers	Covariate Corrections	Marker Exceptions *	Filters ^#^
MUT/Cbl AB	Increasing Weighted Regular Plot differentiators Guidelines: Default %iles	High Markers C3/(C0+C2) C3/C2 C3/C0	Age BW Location	-	-
		Low Markers C18:1/C3 C4/C3			
Cbl CD	Increasing Weighted Regular Plot differentiators Guidelines: Default %iles	High Markers C3/(C0+C2) C3/C2 C3/Met (C3/C2)/(Met*100)	Age BW Location	-	-
		**Low Markers** C18:1/C3			

**Table 3:** Second-tier test for MMA in the Dutch NBS program: outcomes in the period between 10 January 2019 and 31 December 2024 and effect of SCT to differentiate cbl CD and mut/cbl AB SCT (retrospective application); an overview of the full list of settings for both “Single Condition Tools” can be found in Table 2 (https://clir.mayo.edu/, accessed on 22 May 2026).

Outcome 2019	Number of Samples (2019–2024)	Condition	
		cbl CD SCT	mut/cbl AB SCT
Negative based on second tier	5193	3956	2341
MMA referrals with genetic follow-up diagnostic information	42	40	30

## Data Availability

The datasets presented in this article are not readily available because the data are part of an ongoing study. Requests to access the datasets should be directed to the corresponding author.

## Supplementary Materials

The following supporting information can be downloaded at: https://www.mdpi.com/article/10.3390/ijns12030069/s1, Figure S1: (A) Scatterplot depicting the C3 and the C3/2 ratio in genetic and acquired forms of MMA detected via the Dutch MMA NBS (2019–2024). (B) Boxplot comparing the covariate corrected reference intervals (based on age, birthweight and location correction) of the C3/C2 ratio for the reference, MUT/Cbl AB, Cbl CD and B12 Def (mat) populations in CLIR. (https://clir.mayo.edu/ accessed on 22 May 2026).

## Abbreviations

The following abbreviations are used in this manuscript:

MMA	Methylmalonic acidemia
NBS	Newborn Screening
CLIR	Collaborative Integrated Laboratory reports
mut	Mutase
cbl	Cobalamin
MCA	Methylcitric acid
DBS	Dried blood spot
DDRMD	Dutch Diagnosis Registration Metabolic Diseases
TC1	Transcobalamin I
mat	Maternal
SCT	Single Condition Tool
