# Supplementary material for: Refining MMA Screening in the Dutch Newborn Screening Program: Lessons from Vitamin B12 Deficiency and Genetic Cases
Source: Int J Neonatal Screen. 2026 Aug 21;12(3):69. doi: 10.3390/ijns12030069 (PMC13510940; doi:10.3390/ijns12030069)
Supplement: Supplementary file 1 [file IJNS-12-00069-s001.zip › IJNS-4368038-supplementary.pdf]

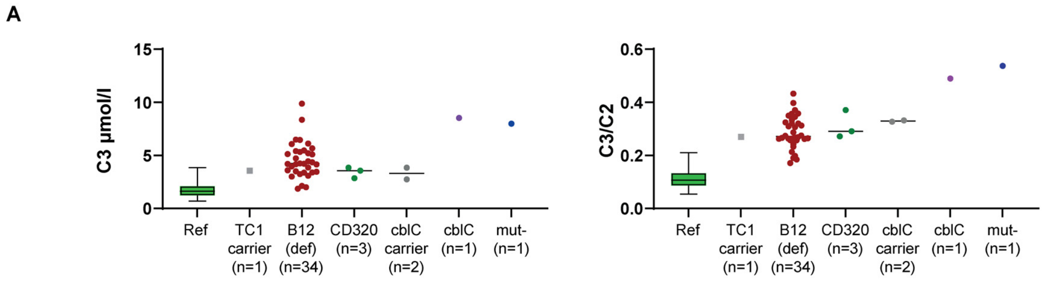

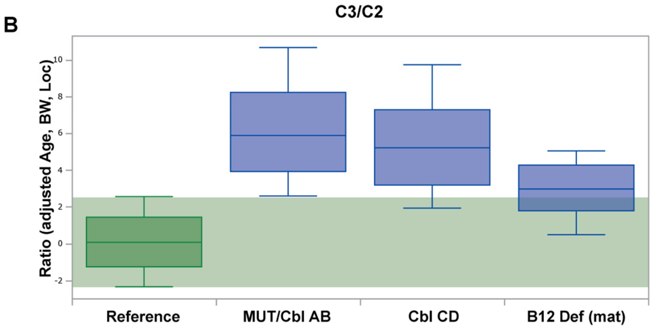

Figure S1. (A) Scatterplot depicting the C3 and the C3/2 ratio in genetic and acquired forms of MMA detected via the Dutch MMA NBS (2019-2024)**. (B)** Boxplot comparing the covariate corrected reference intervals (based on age, birthweight and location correction) of the C3/C2 ratio for the reference, MUT/Cbl AB, Cbl CD and B12 Def (mat) populations in CLIR. (https://clir.mayo.edu/ accessed on the 22th of may 2026).
